# The neural correlates of childhood maltreatment and the ability to understand mental states of others

**DOI:** 10.1080/20008198.2016.1272788

**Published:** 2017-02-09

**Authors:** Charlotte C. van Schie, Anne-Laura van Harmelen, Kirsten Hauber, Albert Boon, Eveline A. Crone, Bernet M. Elzinga

**Affiliations:** ^a^ Institute of Psychology, Faculty of Social Sciences, Leiden University, Leiden, The Netherlands; ^b^ Leiden Institute for Brain and Cognition, Leiden, The Netherlands; ^c^ Department of Psychiatry, University of Cambridge, Cambridge, UK; ^d^ De Jutters Youth Mental Health Care Center, The Hague, The Netherlands; ^e^ Lucertis Child and Adolescent Psychiatry, Rotterdam, The Netherlands; ^f^ Department of Child and Adolescent Psychiatry, Curium-Leiden University Medical Center, Leiden, The Netherlands

**Keywords:** Emotional abuse, emotional neglect, sexual abuse, mentalization, functional imaging, left inferior frontal gyrus, reading the mind in the eyes, emotion understanding

## Abstract

**Background**: Emotional abuse and emotional neglect are related to impaired interpersonal functioning. One underlying mechanism could be a developmental delay in mentalizing, the ability to understand other people’s thoughts and emotions.

**Objective**: This study investigates the neural correlates of mentalizing and the specific relationship with emotional abuse and neglect whilst taking into account the level of sexual abuse, physical abuse and physical neglect.

**Method**: The RMET was performed in an fMRI scanner by 46 adolescents (Age: *M* = 18.70, *SD* = 1.46) who reported a large range of emotional abuse and/or emotional neglect. CM was measured using a self-report questionnaire (CTQ).

**Results**: Neither severity of emotional abuse nor neglect related to RMET accuracy or reaction time. The severity of sexual abuse was related to an increased activation of the left IFG during mentalization even when controlled for psychopathology and other important covariates. This increased activation was only found in a group reporting both sexual abuse and emotional maltreatment and not when reporting isolated emotional abuse or neglect or no maltreatment. Functional connectivity analysis showed that activation in the left IFG was associated with increased activation in the right insula and right STG, indicating that the IFG activation occurs in a network relevant for mentalizing.

**Conclusions**: Being sexually abused in the context of emotional abuse and neglect is related to an increase in activation of the left IFG, which may indicate a delayed development of mirroring other people’s thoughts and emotions. Even though thoughts and emotions were correctly decoded from faces, the heightened activity of the left IFG could be an underlying mechanism for impaired interpersonal functioning when social situations are more complex or more related to maltreatment experiences.

## 1. Background

Childhood maltreatment (CM) is associated with poorer performance on various aspects of social functioning, especially emotion understanding (Luke & Banerjee, 2013). Emotion understanding is an element of mentalizing, the ability to understand and differentiate the mental states of oneself and others (Bateman & Fonagy, 2012; Luyten & Fonagy, 2015). The development of mentalizing is assumed to emerge in interaction with significant others where a child feels emotionally supported (Edwards, Shipman, & Brown, 2005) whereas this development can be hampered when caregivers are consistently unavailable or abusive (Fonagy, Gergely, & Target, 2007; Hildyard & Wolfe, 2002).

Lack of emotional support may be especially experienced by children who have been emotionally abused, a form of CM where a child is often criticized and rejected by parents, or emotionally neglected, in which case parents are emotionally unavailable and unresponsive to a child’s needs and desires (Hornor, 2012). The poorer social functioning that is associated with emotional abuse and neglect (Shaffer, Yates, & Egeland, 2009) could be due to the negative effects emotional maltreatment may have on the development of mentalization skills needed for adaptive interpersonal functioning (Cicchetti, Rogosch, Maughan, Toth, & Bruce, 2003; Edwards et al., 2005; Shipman, Edwards, Brown, Swisher, & Jennings, 2005).

Emotional abuse and neglect are very prevalent, and often co-occur, with other forms of CM, including sexual or physical maltreatment (Hornor, 2012). While the adverse consequences of emotional maltreatment are clearly established (Spinhoven et al., 2010; Spinhoven, Elzinga, Van Hemert, De Rooij, & Penninx, 2016; van Harmelen, 2013; van Harmelen et al., 2014), the specific impact on the development of mentalization remains under investigated (Egeland, 2009; Gilbert et al., 2009; van Harmelen, 2013). Moreover, many studies are based on self-report which can only capture processes people are aware off and willing to report about. More detailed information on the neural processes involved can provide important additional insights into underlying mechanisms associated with the impairments in mentalization related to a history of maltreatment.

Neuroimaging studies have identified a network of brain areas that are particularly relevant for mentalization which can be divided into two processes reflected in subnetworks of the brain (Blakemore, 2008; Herbet et al., 2014; Schurz, Radua, Aichhorn, Richlan, & Perner, 2014). The mirror system refers to the ability to feel what someone else feels by mirroring their inner mental states, and involves a frontoparietal network with the IFG as core structure (Frith & Frith, 2006; Herbet et al., 2014; Marsh & Hamilton, 2011; Shamay-Tsoory, Aharon-Peretz, & Perry, 2009). The network involved in inferring motives and intentions behind inner states is coined the mentalization system per se or perspective taking. This network has the mPFC, pSTS and TPJ as core structures and overlaps with the default mode network (Frith & Frith, 2006; Marsh & Hamilton, 2011; Shamay-Tsoory et al., 2009).

In this study, we are interested in how emotional maltreatment may affect the ability to feel what someone else feels. Facial expressions are an important source of information to mirror inner states of others (Frith & Frith, 2006). The Reading the Mind in the Eyes Task (RMET) uses facial expressions as stimuli and recent findings indicate that the RMET specifically assesses the ability to recognize emotions (Oakley, Brewer, Bird, & Catmur, 2016). We therefore use the RMET to assess the influence of emotional maltreatment on emotion understanding through the mirror system. One study in healthy subjects found that interpersonal stress impaired performance on the RMET and decreased activity in the left IFG, pSTS and TPJ compared to a general stress induction (Nolte et al., 2013), suggesting that acute interpersonal stress can interfere with the neural processes of mirroring and perspective taking.

## 2. Objective

This study aims to expand our knowledge regarding the specific behavioural and neural correlates between emotional abuse and neglect and emotion understanding, using the RMET (Baron-Cohen, Wheelwright, Hill, Raste, & Plumb, 2001) in a sample of adolescents to young adults who experienced various levels of self-reported emotional abuse and emotional neglect. We hypothesized that participants with more severe histories of self-reported emotional abuse and/or neglect show greater difficulties in correctly identifying the mental states of the eyes. Furthermore, we hypothesized that emotional abuse and neglect is associated with differential activation in brain areas involved in the mirror system and mentalization in general: the bilateral IFG, left insula, left cingulate gyrus and bilateral middle temporal gyrus (including the TPJ) (Schurz et al., 2014). We recruited participants based on the reported experience of emotional abuse and/or neglect to ensure a wide range of emotional maltreatment experiences. Because of the intrinsic overlap between emotional abuse and neglect and other forms of maltreatment (i.e. sexual abuse, physical abuse and physical neglect), we will (1) control for these comorbid forms of CM. Moreover, because of the fact that other forms of CM may also affect emotion understanding (Luke & Banerjee, 2013) we will (2) simultaneously investigate the potential impact of these other forms of maltreatment on mirroring mental states. Finally, as the mirror and mentalization system involve a network of brain regions, we should understand neural findings in the context of a larger network (Bressler & Menon, 2010; Pessoa, 2014). We will use functional connectivity analyses to contextualize these findings and to assess whether the severity of maltreatment also affects the connectivity patterns within these networks.

## 3. Method

### 3.1. Participants

A total of 46 adolescents to young adults (Females *N* = 34 and Males *N* = 12, Age range 16–21 years, *M* = 18.70, *SD* = 1.46) participated in this study. This sample covers a wide range (none to severe) of emotional abuse (*M* = 9.04, *SD *= 4.51, range = 5–22) and emotional neglect (*M *= 12.93, *SD *= 5.86, range = 5–24), see Table 1 (Bernstein & Fink, 1998). In addition, 20 participants reported at least one other form of maltreatment: physical abuse (*N *= 5), physical neglect (*N *= 10), sexual abuse (*N *= 13).

**Table 1.** Self-report of childhood trauma (*N*=46). Counts are overlapping and not unique counts.

CTQ	Mean (SD)	#None	#Low	#Moderate	#Severe
Total	40.70 (14.35)				
EA	9.04 (4.51)	23	4	9	10
EN	12.93 (5.86)	13	3	6	24
PA	5.70 (1.71)	41	5	0	0
PN	7.50 (2.78)	36	9	1	0
SA	5.70 (3.32)	33	2	6	5

**Table 2.** Presence of Psychopathology (*N*=46). Counts are overlapping and not unique counts.

Psychopathology	Frequency	Psychopathology	Frequency
*Axis-I disorders Current (#)*		*Lifetime (#)*	
-Major Depression	15	-Major Depression	8
-Social Phobia	7	-Social Phobia	4
-Specific Phobia	2		
-Obsessions	2	-Obsessions	1
-Compulsions	4		
-PTSD	10	-PTSD	2
-Generalized anxiety disorder	1	-Alcohol	3
		-Drugs	4
		-Eating disorder	3
		-Somatization disorder	1
		-Identity Disorder	1
		-ADD	1
*Axis-II disorders (#)*		*VKP (frequency meeting criteria)*	
-Paranoid	4	-Paranoid	12
-Schizoid	1	-Schizoid	10
-Schizotypal	0	-Schizotypal	3
-Antisocial	0	-Antisocial	1
-Borderline	6	-Borderline	8
-Theatrical	0	-Theatrical	0
-Narcissistic	0	-Narcissistic	0
-Avoidant	11	-Avoidant	20
-Dependent	1	-Dependent	7
-Obsessive	2	-Obsessive	4
-Passive-aggressive	1	-Passive-aggressive	1
-Depressive	13	-Depressive	13

**Table 3.** Results of fMRI analyses on RMET using the contrast mental state > gender and FWE cluster extent threshold at *p* < .001 (IFG = Inferior frontal gyrus, STG = Superior temporal gyrus, MTG = Middle temporal gyrus).

		Cluster size		Voxel test value	MNI coordinates		
Analysis	Relation/predictor	K	Label peak voxels	T	X	Y	Z
*One sample* t*-test*							
	Positive	460	L IFG	9.13	−48	29	−2
			L IFG	8.19	−51	14	19
			L IFG	8.05	−54	23	7
		851	L STG	7.90	−60	−52	16
			L STG	7.82	−48	−58	13
			L STG	7.62	−36	−55	19
		416	R STG	6.45	63	−49	13
			R STG	5.55	54	−61	13
			R STG	5.36	54	−40	4
Regression analysis with all CTQ subscales*							
	Sexual Abuse Positive	22	L IFG	4.10	−45	26	−11
			L IFG	3.81	−36	29	−8
PPI analysis**							
	Positive	20	R MTG	4.37	51	−58	4
		35	R Insula	4.26	45	−13	10
		14	R STG	4.04	45	−10	−14
		12	R Parahippocampal gyrus	3.89	18	−43	−2

*Masked by whole brain mental state > gender contrast, ** cluster extent threshold with *p *< .001 and 10 voxels.

Twenty-three participants (Females *N* = 18 and Males *N* = 5, Age *M* = 18.17, *SD* = .98) were recruited from a mental health institution in The Hague, the Netherlands, who reported at least mild to moderate emotional abuse and/or neglect.^1^ These participants also participated in a social exclusion study described in van Harmelen et al. (2014). Furthermore, 23 participants (Females *N* = 16 and Males *N* = 7, Age *M* = 19.22, *SD* = 1.68) were recruited from the general population and matched for age and gender (see Gunther Moor et al. (2012) (*N *= 18) and van Harmelen et al. (2014) (*N *= 5)). There was no restriction on level of emotional maltreatment or psychopathology so that the total group (*N* = 46) would cover a wide range of emotional maltreatment including a middle range. Inclusion criteria were the same for all participants and were right handedness, understanding the Dutch language, being able to provide informed consent, MRI compatibility, no substance abuse or psychosis. Due to technical MRI problems data of seven participants were discarded (*N* = 2 from the general population), resulting in the described sample (*N* = 46).

IQ was estimated using the subtests Similarities and Block design of the WAIS (≥ 17 years) or the WISC (16 to 17 years) (Wechsler, 1991, 1997). The sample had an average IQ, *M* = 108.36, *SD* = 9.41. Treatment duration for participants from the mental health institution ranged from 0 to 17 months (*Md* = 2.00, *M* = 4.30, *SD* = 5.04). Eleven participants took a stable dose of psychiatric medication (SSRIs (*N* = 8), Benzodiazepine (*N* = 2) and Amitriptyline (*N* = 1)). Most common Axis-I disorders were major depression and PTSD, see Table 2. Nine participants reported one Axis-I disorder and four participants reported two or more Axis-I disorders. Most common personality disorders (PD) were depressive and avoidant PD, see Table 3. Ten participants had one PD, and 11 participants had two or more PDs.

The informed consent was signed by the participant as well as a parent or legal guardian when participants were under the age of 18. The study was approved by the Leiden University Medical Center Medical Ethics Committee and was conducted in accordance with the declaration of Helsinki and the Medical Research Involving Human Subjects Act (WMO).

### 3.2. Materials

#### 3.2.1. Childhood Trauma Questionnaire – Short Form (CTQ-SF)

All participants completed the Dutch version of 28-item CTQ-SF which measures the severity of CM, and includes five subscales: emotional abuse (EA), emotional neglect (EN), physical abuse (PA), physical neglect (PN), and sexual abuse (SA) (Arntz & Wessel, 1996; Bernstein et al., 2003). The questionnaire has been validated in the Dutch population (Thombs, Bernstein, Lobbestael, & Arntz, 2009). Each subscale is measured with five 5-point scale items except for the SA scale which consisted of four items (item SA21 was removed, see Thombs et al., 2009). The subscale scores are calculated by summing over the items and could range from 5–25 or in the case of SA 4–20.

The internal consistency was good for the following subscales; EA = .88, EN = .93, SA = .91. The internal consistency of physical abuse was reasonable (PA = .64) and poor for physical neglect (PN = .47). This is in line with other studies. The correlation between EA and EN is strong, *r* = .59, however, there were no statistical problems related to multicollinearity (all VIF < 1.83).

#### 3.2.2. Measures of psychopathology

To assess lifetime and current Axis-I psychopathology participants at the mental health institution were examined with the SCID-I administered by a trained psychology student (C.v.S) (First, Spitzer, Gibbon, & Williams, 1997). Further, all participants were assessed on pathological personality traits using the questionnaire on Personality Traits (Dutch: Vragenlijst voor Kenmerken van de Persoonlijkheid (VKP)) which is based on the semi-structured interview IPDE and assesses the degree of presence of 12 PDs according to DSM-IV and ICD-10. The VKP consists of 197 items rated on a 3-point scale (True, ?, and False) and was used as a screening instrument for Axis-II disorders (Duijsens, Eurelings-Bontekoe, & Diekstra, 1996). The SCID II, a semi-structured interview based on DSM-IV, was used to assess the presence of an Axis-II personality disorder indicated by the VKP as ‘probable’ or ‘present’ (Duijsens et al., 1996). The Dutch translation was administered by a clinical psychologist (K.H.) (First, Gibbon, Spitzer, Williams, & Benjamin, 2000; Weertman, Arntz, Dreessen, van Velzen, & Vertommen, 2003).

#### 3.2.3. Reading the Mind in the Eyes Task

The RMET task, which was adapted for fMRI purposes (see Gunther Moor et al., 2012), consists of two types of trials, mental state and age/gender trials (hereafter gender trials), depicting photos of the eye region of faces. During the mental state trials participants indicated what the depicted person is thinking or feeling. Participants responded by choosing one of four given answers. During the gender trials participants indicated the gender (male vs female) and age (young (60-) vs old (60+). The location of the four given answers varied on trial basis. The correct responses on three gender trials were evaluated by gender only due to low agreement on age in these pictures (see Gunther Moor et al., 2012).

The task consists of two blocks of 14 trials per condition, resulting in 56 trials in total. Each eye region photo appeared in the mental state as well as the gender condition. The blocks were presented alternately and the type of starting block was counterbalanced among participants. Each trial started with a fixation cross which was jittered with a pseudorandom duration of 600–8000 ms. The stimulus with four answers was shown for 8000 ms regardless of how fast participants responded. Failing to answer within 8000 ms evoked a feedback screen with the message ‘too slow’ (0.08% of gender trials and 1.16% of mental state trials). Participants were familiarized with the MRI scanner using a mock scanner and introduced to the task using four example trials.^2^

### 3.3. Data analysis

To study a dose-response effect of EA and EN severity on emotion understanding, we investigated in the total sample the impact of EA and EN as continuous predictors. Because of the substantial overlap with other forms of CM, and to investigate whether physical maltreatment and/or sexual abuse had an independent effect on emotion understanding, we took PA, PN and SA into account in the analyses. Furthermore, we checked for the influence of potential confounding variables: age (in years), gender, level of psychopathology (total VKP score), medication status (on or off), treatment duration (in months) and IQ (Baker, Peterson, Pulos, & Kirkland, 2014). Even though there was no relation between age, gender, IQ and treatment duration on the one hand and EA and EN on the other hand, we checked whether any of the hypothesized results could not be explained by these variables. Medication status was positively related to both EA (*r *= .44) and EN (*r *= .51).

#### 3.3.1. Behavioural data analysis

RMET performance was analysed with IBM SPSS Statistics version 23 using repeated measures ANOVA with number of correct responses or reaction time as outcome variable, condition (mental state or gender trial) as within-subjects factor and the CTQ subscales as continuous predictors. To correct for multiple testing alpha was Bonferroni corrected: .05/2 outcomes (RTs and correct responses) = .025.

#### 3.3.2. MRI acquisition

Scans were acquired using a 3.0 Tesla Phillips MRI scanner. A survey scan was conducted followed by a T2*-weighted Echo Planar Images (EPI) functional MRI (fMRI) scan during the RMET task. The scan session finished with a high resolution T1-weighted anatomical scan. The scan parameters used during the T2*-weighted EPI functional MRI scan were as follows: TR = 2200 ms, TE = 30 ms, slice matrix = 80 x 80, number of slices per volume = 38, FOV = 220 mm (ap), 114.675 mm (fh), 220 mm (rl). T1-weighted structural images were recorded with the following FOV: 224 mm (ap), 168 mm (fh) and 177.333 mm (rl).

#### 3.3.3. fMRI data preprocessing

Data was preprocessed in Statistical Parametric Mapping version 8 (SPM8; Wellcome Department of Cognitive Neurology, London, UK). Motion correction was applied. No subject moved more than 3 mm (max movement = 1.56 mm) and mean movement was not related to any form of CM (all *r*s < |.26|, all n.s.). Slice time correction was applied. A high pass filter of 120 Hz was used. Functional images were registered to their respective T1 scan before being normalized to a template based on the MNI152 stereotaxic space. The normalization was achieved using a 12-parameter affine transformation followed by a non-linear transformation based on the cosine transform basis function. After normalization, voxel size was 3 × 3 × 3 mm. Data were spatially smoothed using an 8 mm FWHM isotropic Gaussian kernel.

#### 3.3.4. fMRI data analysis

Data analysis was performed in SPM8. All trials were modelled regardless of whether they were answered correctly. Trials in which participants did not respond within 8000 ms were seen as invalid and therefore not analysed. The duration of the trials was modelled as the length of the reaction time (Gunther Moor et al., 2012). For all analyses, we used cluster extent thresholding with *p *< .001 and FWE cluster extent size (Woo, Krishnan, & Wager, 2014). To investigate the functional activity related to the mirror system, we performed a one-sample *t*-test on the mental state > gender contrast, in the whole group on a whole brain level.

To investigate the influence of EA, EN, SA, PA and PN on neural activation, we performed a regression analysis with all five CTQ subscales as predictors in the mental state > gender contrast, testing for the negative and the positive relation with neural activation. We masked this regression analysis with the result of the whole brain mental state > gender contrast to only examine areas related to mental state activation.

To yield a better interpretation of the areas where significant dose-response associations with CM are found, functional connectivity analyses were used to examine the functional relations between these significant areas and other brain areas in the mental state > gender contrast using PsychoPhysiological Interaction (PPI) analyses (gPPI Toolbox; McLaren, Ries, Xu, & Johnson, 2012). To examine which functional relations between areas were stronger in the mental state condition than the gender condition, we performed a whole group, whole brain one sample *t*-test. Furthermore, we performed a regression analysis with the CTQ subscales related to neural findings as continuous predictors to investigate whether there was differential connectivity based on CM.

## 4. Results

### 4.1. Behavioural results – RMET performance

The RM ANOVA with all five CTQ subscales as predictors showed that, overall, the gender trials were answered more often correctly (*M*_gender_ = 24.59, SE = .37; *M*_mental state_ = 19.28, SE = .34) and faster (*M*_gender_ = 2.77, SE = .07; *M*_mental state_ = 4.15, SE = .10) than the mental state trials, Accuracy: *F*(1,40) = 10.72, *p* = .002, *r* = .46; Reaction Time (RT): *F*(1,40) = 14.72, *p* < .001, *r* = .52. None of the CTQ subscales showed a relation to the number of correct trials or reaction time on the trials. Furthermore, there were no interaction effects between the CTQ subscales and type of trial on the number of correct trials or on reaction time.

### 4.2. Neural results – RMET activation

#### 4.2.1. Whole brain mental state > gender contrast

**Figure 1.**
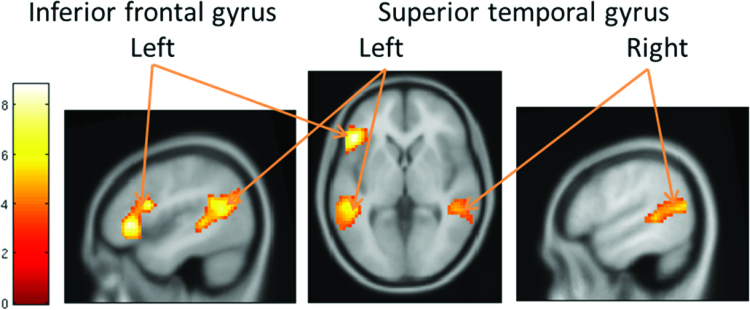
Whole brain one sample *t*-test on mental state > gender contrast. FWE cluster extent threshold with *p *< .001, left = left, MNI −45, 29, 1 (left and middle), MNI 54, −31, 13 (right).

A whole brain one sample *t*-test analysis on the mental state > gender contrast revealed three clusters: one in the left IFG, one in the left superior temporal gyrus and one in the right superior temporal gyrus, see Table 3 and Figure 1. These results are in line with findings from other RMET studies (Schurz et al., 2014).

#### 4.2.2. Effect of childhood maltreatment on mentalizing

**Figure 2.**
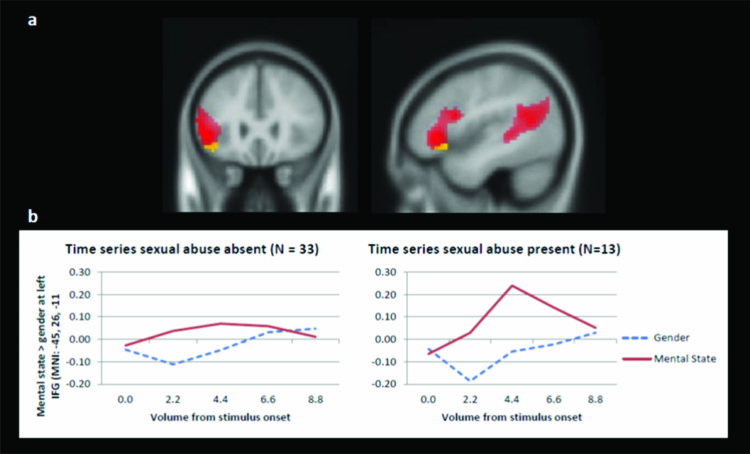
a: Positive relation between SA and left IFG (MNI: −45, 26, 11) resulting from a regression analysis with all subscales (Yellow, FWE cluster extent threshold with *p *< .001) depicted on whole brain mental state > gender contrast (Red, FWE cluster extent with *p *< .001). b: Time series of gender and mental state contrast represent the mean activation of the active cluster in the left IFG (MNI: −45, 26, −11) for five volumes (TR = 2.2 s) starting at stimulus onset to four volumes after. The time series covers one trial (8000 ms) and the onset of the next trial (800 ms).

A regression analysis with all five CTQ subscales as predictors on the mental state > gender contrast revealed neither an effect for EA nor EN. Rather, a significant positive relation was observed between SA and neural activation in the left IFG, see Table 3 and Figure 2(a). The time series in Figure 2(b) indicate that in individuals reporting SA (*N* = 13, low to severe SA) the left IFG is more active during mental state trials compared to the gender trials whereas this increase in activation is less pronounced in individuals not reporting SA (*N* = 33, no SA) (Bernstein & Fink, 1998).

#### 4.2.3. Effect of confounding variables

Age, gender, IQ, psychopathology and medication status did not change the results of the effect of SA on differential neural functioning of the left IFG during mental state > gender contrast. Including treatment duration in the regression analysis reduced the effect of SA on the left IFG, where it remained significant at *p* < .001 and extent threshold of 10 voxels. In a subsample only (*N* = 27), state dissociation as measured by the DSS-4 has been taken into account as well and did not change the effect of SA on the left IFG at *p* < .001 and an extent threshold of 10 voxels.

#### 4.2.4. Exploratory follow-up analysis

It must be noted that all participants reporting a history of SA also reported high levels of EA and/or EN. Of the 13 participants reporting SA, 12 participants also reported moderate to severe EA and/or EN and one participant reported low EN (Bernstein & Fink, 1998). Since statistically controlling for SA, EA or EN is not sufficient, we set off to elucidate the effects of SA in the context of childhood emotional maltreatment (CEM: EA and/or EN) and the effect of CEM without concurrent SA on the activation in the left IFG. We used an exploratory ANOVA comparing three groups: one control group (reporting no SA and no CEM, *N* = 13), one group reporting isolated CEM (low to severe levels of CEM & no SA, *N* = 20) and one group reporting both CEM and SA (low to severe CEM & low to severe SA, *N* = 13) (Bernstein & Fink, 1998). We used ROI analysis to extract the individual contrast values of the mental state > gender contrast of the left IFG cluster. In SPSS (version 23), a one-way ANOVA was performed with three a priori contrasts: no abuse vs isolated CEM, no abuse vs CEM and SA, and isolated CEM vs CEM and SA. The groups differed in left IFG activation, *F*(2,45) = 6.24, *p* = .004. Conservative *t*-contrasts indicate that the group reporting both CEM and SA showed increased activation of the left IFG compared to both the no abuse group, *t*(15.19) = 2.91, *p* = .01 and the isolated CEM group, *t*(17.53) = 2.40, *p* = .03. There was no difference between the no abuse and the isolated CEM group, *t*(30.97) = .74, *p* = .47, suggesting that the combination of SA and CEM is related to increased neural activity in the left IFG during mentalization and not CEM alone.

#### 4.2.5. Functional connectivity analysis

**Figure 3.**
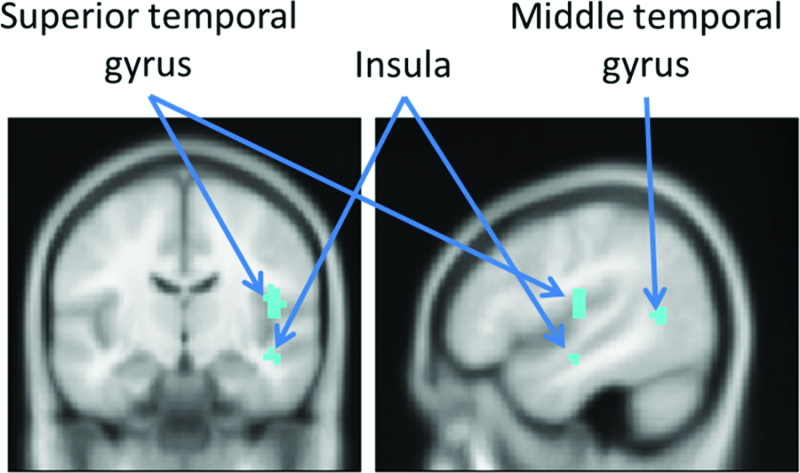
Results of functional connectivity analysis from cluster in left IFG related to SA, cluster extent threshold of 10 voxels and *p* < .001, MNI: 47, −12, 18, right = right.

The PPI analysis, extent thresholded with 10 voxels and *p *< .001, with the significant cluster in the left IFG revealed functional connections to activity in the right superior temporal gyrus, the right middle temporal gyrus and the right insula and the right parahippocampal gyrus for the mental state > gender contrast, see Table 3 and Figure 3 (Mars et al., 2012). No differential functional connectivity was found based on the level of reported SA.

## 5. Discussion

In this study we investigated whether emotional abuse and emotional neglect as well as other forms of CM are related to difficulties in mentalization during the RMET task, using behavioural as well as neural data. We found that emotionally abused and neglected adolescents do not show impairments in mentalizing in terms of their performance on the RMET. This contradicts findings of impairments in emotion understanding in emotionally maltreated children (Edwards et al., 2005; Luke & Banerjee, 2013). However, differences in emotion understanding among maltreated children tend to disappear in adolescence and adulthood (Luke & Banerjee, 2013). This could indicate that at an older age developmental delays of the more basic skill of emotion understanding are overcome perhaps by the use of different strategies. Alternatively, impaired mentalizing at this age may be observable only in more complex or stressful situations (Nazarov et al., 2014) and/or by other means of measurement such as neurobiological measures.

Interestingly, on the neural level, we found that not the level of emotional abuse or emotional neglect but the severity of sexual abuse was associated with an increased activation in the left IFG during mentalization. This effect could not be explained by age, gender, IQ, medication status or level of psychopathology. Furthermore, this effect seemed specific to participants who had experienced both emotional maltreatment and sexual abuse but not emotional maltreatment alone.

The left IFG is important in the mentalization process and is thought to be involved in emotion understanding through the mirror system (Carr, Iacoboni, Dubeau, Mazziotta, & Lenzi, 2003; Keysers, Kaas, & Gazzola, 2010; Schurz et al., 2014; Shamay-Tsoory et al., 2009). In healthy subjects performing the RMET, the bilateral IFG was recruited more by younger (10–12 years) than older adolescents (14–23 years) indicating an automation of the mirroring process over time (Gunther Moor et al., 2012; Overgaauw, van Duijvenvoorde, Gunther Moor, & Crone, 2015). The increased activation of the left IFG related to sexual abuse may therefore indicate a less developed mirroring system. Considering the network perspective of mentalizing, we found regions functionally related to the left IFG, i.e. STG, MTG, parahippocampal gyrus and insula, that are part of the mentalization network and overlaps with the default mode network (Herbet et al., 2014; Mars et al., 2012; Spreng, Mar, & Kim, 2009). The connectivity within this network did not vary with levels of sexual abuse. Thus, the activation in the left IFG is likely related to part of the mentalization network, however, it is the activity of the left IFG and not the connectivity within this network that is related to sexual abuse. Together, these findings indicate that being both sexually abused and emotionally maltreated may lead to the use of a different strategy or delayed development in mirroring mental states requiring a stronger involvement of the left IFG. Even though the behavioural performance on the RMET was not affected by maltreatment history, the increased neural activation of the left IFG may be an interesting basis for the underlying mechanism of how the ability to mentalize may be altered.

To the best of our knowledge, relatively few studies investigated the specific link between sexual abuse and mentalization and reported some evidence for impaired mentalizing (Dauphin, Lecomte, Bouchard, Cyr, & David, 2013; Lysaker et al., 2011; Mielke et al., 2016). Interestingly, several studies from the 1990s have shown that negative outcomes of sexual abuse, among which is impaired social functioning, are not only related to characteristics specific to sexual abuse itself, but that the familial context, particularly maternal warmth, maternal availability and parental support by the non-abusive parent, plays an important role as well (Alexander, 1992; Cole & Putnam, 1992; Harter, Alexander, & Neimeyer, 1988; Polusny & Follette, 1995). This is exactly what is being omitted in the case of emotional neglect and abuse (Egeland, 2009; Hornor, 2012). The combination of sexual abuse and parental emotional maltreatment may therefore be particularly toxic. This offers an interesting perspective on our findings that the combination of sexual abuse and emotional maltreatment (and not emotional maltreatment alone) was related to enhanced neural activation of the left IFG when mirroring mental states.

Strengths of this study are that all maltreatment types have been studied simultaneously, that we recruited a sample with a large range of emotional maltreatment severity and that potential confounding variables have been taken into account. Furthermore, the findings related to the neural processes in the brain during mentalizing yield new insights into the possible mechanisms of hampered mentalization. This study also has its limitations. CM was measured retrospectively using a self-report questionnaire, which could be sensitive to subjectivity and recall bias. However, studies have shown that retrospective measures of CM are reliable (Hardt & Rutter, 2004; Tonmyr, Draca, Crain, & Macmillan, 2011). Future studies should use a longitudinal design to provide better insights into whether CM delays the development of mentalization. Furthermore, it should be noted that the absence of effects of the physical abuse and neglect subscales may be due to the low reliability of both scales as well as the low prevalence of reported physical abuse. With respect to the lack of associations between maltreatment and performance on the RMET task, it should be noted that the RMET may not be sensitive enough to pick up on differential mentalization ability (Oakley et al., 2016). It can be argued that more complex social tasks or stimuli with higher self-relevance or emotional intensities should be used (Nazarov et al., 2014; Nolte et al., 2013). As this study was designed to investigate the link between emotional abuse and neglect and we therefore recruited participants based on emotional maltreatment histories, we could not disentangle the specific effect of sexual abuse with and without emotional maltreatment on mentalization. We did however find that emotional maltreatment alone was not related to alterations in mentalization. Future studies should simultaneously study the various types of maltreatment and the parenting context in which the maltreatment has taken place to be able to understand the unique but also combined effects of CM experiences on social cognition (Teicher, Samson, Polcari, & McGreenery, 2006). In this case, statistical control is not sufficient (Briere & Elliot, 1993).

## 6. Conclusions

Individuals reporting sexual abuse as well as emotional maltreatment show an altered and/or delayed mirroring of other people’s emotions as indicated by the stronger involvement of the left IFG during mentalization. This could potentially be an underlying mechanism for impaired interpersonal functioning in more complex or more personally relevant social situations.

## Supplementary Material

Supplementary material

## Notes

1. See **Supplementary Table 1.** For descriptive purposes only, we provided the descriptive statistics of the CTQ subscales split by participants recruited from the general population and mental health institution.

2. Note that participants performed RMET before being introduced to and playing the Cyberball game as reported in van Harmelen et al. (2014).
