# Supplementary material for: The neural correlates of childhood maltreatment and the ability to understand mental states of others
Source: Eur J Psychotraumatol. 2017 Feb 9;8(1):1272788. doi: 10.1080/20008198.2016.1272788 (PMC5328315; doi:10.1080/20008198.2016.1272788)
Supplement: Supplementary material [file zept_a_1272788_sm6406.pdf]

Supplementary Material

Supplementary Table 1. CTQ subscales for participants recruited from the general population and mental health institute

	General Population sample (N=23)				Patient sample (N=23)				Difference
	Mean	Std. Dev.	Min	Max	Mean	Std. Dev.	Min	Max	p-value
CTQ EA	5.61	1.31	5	10	12.48	3.90	6	22	<.001
CTQ EN	8.83	4.32	5	19	17.04	4.03	10	24	<.001
CTQ PA	5.30	1.46	5	12	6.09	1.88	5	11	.122
CTQ PN	5.96	1.72	5	12	9.04	2.80	5	16	<.001
CTQ SA	4.04	.21	4	5	7.35	4.10	4	15	<.001
